# Fc-independent functions of anti-CTLA-4 antibodies contribute to anti-tumor efficacy

**DOI:** 10.1007/s00262-022-03170-z

**Published:** 2022-03-03

**Authors:** Yosuke Sato, Cierra N. Casson, Atsushi Matsuda, James I. Kim, Judy Qiuju Shi, Shinji Iwasaki, Susan Chen, Brett Modrell, Chingkit Chan, Daniel Tavares, Douglas Austen, Koh Ida, Olga Tayber, Pyae Hein, Robert Comeau, Yafang Lin, Michael H. Shaw

**Affiliations:** 1 Takeda Pharmaceuticals International Co., Cambridge, MA USA; 2 Mediar Therapeutics, Cambridge, MA USA; 3 Dragonfly Therapeutics, Waltham, MA USA; 4 Curia, Hopkinton, MA USA

**Keywords:** Anti-CTLA-4 antibody, Fc effector functions, Anti-tumor efficacy, Syngeneic tumor model

## Abstract

Ipilimumab, a monoclonal antibody that recognizes cytotoxic T-lymphocyte associated protein 4 (CTLA-4), was the first immune checkpoint inhibitor approved by the FDA to treat metastatic melanoma patients. Multiple preclinical studies have proposed that Fc effector functions of anti-CTLA-4 therapy are required for anti-tumor efficacy, in part, through the depletion of intratumoral regulatory T cells (Tregs). However, the contribution of the Fc-independent functions of anti-CTLA-4 antibodies to the observed efficacy is not fully understood. H11, a non-Fc-containing single-domain antibody (VHH) against CTLA-4, has previously been demonstrated to block CTLA-4-ligand interaction. However, in vivo studies demonstrated lack of anti-tumor efficacy with H11 treatment. Here, we show that a half-life extended H11 (H11-HLE), despite the lack of Fc effector functions, induced potent anti-tumor efficacy in mouse syngeneic tumor models. In addition, a non-Fc receptor binding version of ipilimumab (Ipi-LALAPG) also demonstrated anti-tumor activity in the absence of Treg depletion. Thus, we demonstrate that Fc-independent functions of anti-CTLA-4 antibodies contributed to anti-tumor efficacy, which may indicate that non-Treg depleting activity of anti-CTLA-4 therapy could benefit cancer patients in the clinic.

**Supplementary Information:**

The online version contains supplementary material available at 10.1007/s00262-022-03170-z.

## Introduction

Immune checkpoint blockade therapies have been approved for multiple cancer indications, because of their durable responses and long-term remissions [1–3]. Despite many preclinical and clinical studies aimed at understanding the underlying biological mechanism of anti-CTLA-4 therapy, the mechanism remains to be fully elucidated [4].

In the process of activation of naïve T cells in response to a specific antigen, at least two signals are required for proper T cell activation [5]. The first signal is recognition and binding of the T cell receptor to antigen-bound major histocompatibility complex (MHC) on the antigen-presenting cells (APCs). The other signal is simultaneous costimulatory engagement of CD28 on the T cells by B7 family members such as B7-1 (CD80) and B7-2 (CD86), on the APCs. CTLA-4 is a homologue of CD28 and binds to B7-1/2 with greater avidity and affinity than CD28 and inhibits interaction between B7 ligands on APCs and CD28 on T cells, resulting in the dampening of T cell activation. The critical role of CTLA-4 in regulating T cell activation is underscored by the enhanced susceptibility to autoimmune diseases observed in patients harboring mutations in CTLA-4. [6, 7]. CTLA-4 is not only expressed on activated CD4 + and CD8 + T cells during the activation phase but is also constitutively expressed on regulatory T cells (Tregs). Specific loss of CTLA-4 in Tregs is sufficient to induce aberrant T cell activation resulting in fatal autoimmunity [8, 9]. This indicates that CTLA4 expression on Tregs is necessary for Tregs to exhibit suppressive function and to maintain immunologic tolerance.

In preclinical studies in murine cancer models, anti-CTLA-4 monoclonal antibodies were initially thought to act simply via blocking CTLA-4 on effector T cells and Tregs [10], but subsequent studies demonstrated that the activity of anti-CTLA-4 antibodies may extend beyond CTLA-4 blockade of effector T cells and Tregs, relying upon concomitant depletion of Tregs for maximal anti-tumor activity. Anti-CTLA-4-mediated anti-tumor activity was positively dependent on Fc-mediated effector functions, such as antibody-dependent cell-mediated cytotoxicity (ADCC) and complement-dependent cytotoxicity (CDC). In the colon cancer models CT-26 and MC38, anti-CTLA-4 mIgG2a antibody with enhanced Fc effector functions promoted enhanced anti-tumor activity through the reduction of intratumoral Tregs, as compared to tumor-bearing animals treated with anti-CTLA-4 antibodies of different isotypes with weaker effector functions, such as mIgG2b or mIgG1, or anti-CTLA-4 antibody lacking Fc effector functions (IgG1-D265A) [11]. Furthermore, in two other distinct syngeneic tumor models, blunted anti-tumor immunity was observed when FcγR-null animals were treated with either anti-CTLA-4 mIgG2b (clone 9D9) alone or in combination with an irradiated B16-BL6 tumor cell-based vaccine that secretes GM-CSF (GVAX). Collectively, these data suggested that selective FcγR-mediated intratumoral Treg depletion was a driver in promoting anti-CTLA-4 antibody-mediated anti-tumor activity [12, 13]. Furthermore, in the B16F10 melanoma tumor model, neither an anti-CTLA-4 VHH single-domain nanobody lacking the Fc-portion nor a pegylated VHH controlled tumor growth in combination with GVAX, while a VHH fusion to mIgG2a restored therapeutic efficacy with reduction of intratumoral Tregs [14]. Taken together, there are strong preclinical data suggesting that intratumoral Treg depletion is a mode-of-action for generating anti-tumor immunity following anti-CTLA-4 therapy.

In the clinic, the mechanism of anti-tumor efficacy of anti-CTLA-4 therapy is still under debate. In an ex vivo co-culture system with monocytes and T cells sorted from human PBMC, ipilimumab depletes Tregs via an ADCC-dependent mechanism mediated by FcγRIIIA (CD16)-expressing non-classical monocytes [15]. Additionally, in patients with advanced melanoma, the response to ipilimumab was associated with the activating FcγR CD16a-V158F high-affinity polymorphism [16]. In contrast, quantitative IHC analysis of tumor tissues from cancer patients shows that anti-CTLA-4 immunotherapy using ipilimumab or tremelimumab (anti-human CTLA-4 hIgG2) did not deplete Foxp3+cells in human tumors [17]. Although tremelimumab treatment did not reach statistical significance in overall survival at the planned second interim analysis in the phase III clinical trial in metastatic melanoma, follow-up analyses suggest that responses to tremelimumab are roughly comparable to those of ipilimumab, which has more Fc effector functions than tremelimumab [18]. These data indicate that not only Fc-dependent effector functions but also Fc-independent functions of anti-CTLA-4 antibodies may contribute to the anti-tumor efficacy in patients.

In the present study, to interrogate the Fc-independent function of anti-CTLA-4 antibodies in promoting anti-tumor activity, we generated two types of anti-CTLA-4 antibodies with or without Fc effector functions. One is a half-life extended non-Fc-containing single-domain antibody (VHH) against CTLA-4, and the other is an Fc mutant of ipilimumab with a mIgG2a isotype. In the present report, we show that anti-CTLA-4 antibodies lacking Fc-mediated effector functions still induce robust anti-tumor activity in preclinical tumor models.

## Material and methods

### Animals

C57BL/6 and BALB/c mice were purchased from Jackson Laboratory and Beijing Vital River Laboratory Animal Technology Co., Ltd. (Beijing, China). Human CTLA-4 knock-in C57BL/6 mice were purchased from Beijing Biocytogen Co., Ltd. (Beijing, China). All studies were conducted under the approval of the Takeda Oncology Institutional Animal Care and Use Committee.

### Antibodies

Anti-mouse CTLA-4 (CD152) mAb 9D9 was purchased from Bio-X Cell Inc. (West Lebanon, NH, the USA).

### Plasmid construction and expression for antibody generation

Amino acid sequences of H11, anti-serum albumin VHH and ipilimumab were obtained from a previous report [14], patent (US20070269422A1, Serum albumin binding proteins with long half-lives) and DrugBank (https://www.drugbank.ca/drugs/DB06186), respectively. H11 DNA was synthesized at Integrated DNA Technologies, Inc. H11-HLE DNA was synthesized at SynBio Technologies. Ipi-WT and Ipi -LALAPG were synthesized at GENEWIZ. All ORFs are shown in Supplementary Figs. 1 and 2. The H11 sequence was cloned into pET22b (Millipore), and the plasmid was grown with TB media. The induction was performed with 1 mM IPTG overnight at 25 °C. The cultured supernatant was harvested by centrifugation. H11-HLE, Ipi-WT and Ipi-LALAPG were cloned into pcDNA3.4 (Thermo Fisher). H11-HLE and Ipi-LALAPG were expressed by Expi293F cells (ThermoFisher), and Ipi-WT was expressed by ExpiCHO (ThermoFisher). All mammalian cell transfections and expression were performed following the manufacturer’s protocols.

### Purification of antibodies

The culture supernatants which contained H11 and H11-HLE were loaded onto cOmplete™ His-Tag Purification Resin (Roche), followed by Capto S (GE Healthcare), Capto Q (GE Healthcare), and Superdex 75 (GE Healthcare). The final concentrated proteins were stored in PBS. The culture supernatants which contained Ipi-WT and Ipi-LALAPG were loaded onto MabSelect SuRe LX (GE Healthcare). The eluted proteins were neutralized with 1 M trisodium citrate. The final concentrated proteins were stored in 25 mM sodium citrate, 125 mM NaCl pH 5.5. All purified proteins were > 95% pure, confirmed by SDS-PAGE. All endotoxin levels were below 0.02 E.U./mg.

### Mouse CTLA-4 binding assay

Binding affinity of anti-mouse CTLA-4 VHHs was evaluated by an enzyme-linked immunosorbent assay (ELISA)-based method. Briefly, recombinant mouse CTLA-4 protein reconstituted in PBS was coated onto 96-well microplates overnight at room temperature. After washing with PBS-T (PBS, 0.05% Tween 20), the plates were incubated with blocking buffer (1 × Blocker BSA in PBS) (Thermo Fisher Scientific) for one hour at room temperature followed by washing with PBS-T. A titrated series of H11 or H11-HLE was added to the plates and incubated for 1.5 h. After washing with PBS-T, each well was incubated for one hour with anti-6xHis tag antibody HRP (Abcam) followed by another wash step. TMB substrate reagent set (BD Bioscience) was then added according to the manufacturer’s protocol. Absorbance of each well at 450 nm was measured by SpectraMax i3x (Molecular Devices) immediately after adding 1 N sulfuric acid (Sigma) as a stop solution.

### Surface Plasmon resonance measurements

SPR experiments to evaluate human CTLA-4 and mouse FcγRIII binding were performed using BIACORE 8 K (GE Healthcare) equipped with Series S CM5 sensor chip. Anti-histidine antibody was immobilized using amine-coupling chemistry. The surfaces of flow cells were activated for 7 min with a 1:1 mixture of 0.1 M NHS (*N*-hydroxysuccinimide) and 0.4 M EDC (3-(*N*,*N*-dimethylamino) propyl-*N*-ethylcarbodiimide) at a flow rate of 10 μl/min. Anti-histidine antibody at a concentration of 25 μg/ml in 10 mM sodium acetate, pH 4.5, was immobilized at a flow rate of 10 μl/min for 10 min on both Fc12 in designated channels. Both surfaces were blocked with a 7 min injection of 1 M ethanolamine, pH 8.0. To collect kinetic binding data, ligand, His-tagged human CTLA-4 or mouse FcγRIII, was captured over Fc2 of designated channels at a flow rate of 10 μl/min and at a temperature of 25 °C. The complex was allowed to associate for 60 s. Ipi-WT or Ipi-LALAPG was injected in Fc12 of the channel at a flow rate of 30 μL/min and at a temperature of 25 °C. The Ipi-WT or Ipi-LALAPG was allowed to associate and dissociate for 60 s and 180 s, respectively. The surfaces were regenerated with a 60 s injection of 10 mM glycine–HCL pH 1.5 at a flow rate of 30 μL/min. Data were collected at a rate of 10 Hz. The data were fit to a 1:1 binding model using the global fit option available within Biacore 8 K Evaluation software.

SPR experiments to evaluate binding to mouse FcγRI and FcγRIV were performed using a BIACORE 8 K (GE Healthcare) equipped with biotin capturing surface sensor chip. Biotin CAPture reagent was immobilized to the sensor chip by hybridization of complementary ssDNA oligo at a flow rate of 2 μL/min for 300 s. To collect kinetic binding data, biotinylated mouse FcγRI or FcγRIV (SinoBiologica) was captured over Fc2 of designated channels at a flow rate of 10 μL/min for 60 s and at a temperature of 25 °C. Ipi-WT or Ipi-LALAPG was allowed to associate and dissociate for 180 s and 600 s, respectively. The surface was regenerated with a 120 s injection of 3:1 8 M Gdn-HCl/1 M NaOH solution at a flow rate of 10 μL/min. Data were collected at a rate of 10 Hz. The data were fit to a 1:1 binding model using the global fit option available within Biacore 8 K Evaluation software.

### Cell culture

The murine colon tumor cell line MC38 was purchased from ATCC (Manassas, VA, the USA) and Obio Technology (Shanghai) Corp., Ltd. (Shanghai, China). H22 was purchased from China Center for Type Culture Collection (Beijing, China). Human PBMC was purchased from STEMCELL Technology. Raji cells expressing an engineered cell surface protein designed to activate cognate TCRs in an antigen-independent manner and endogenously expressing CTLA-4 ligands CD80 and CD86 were purchased from Promega Corporation (Madison, WI, the USA). All cell lines were incubated at 37 °C and maintained in an atmosphere containing 5% CO_2_. MC38 cells were grown in DMEM (Gibco) supplemented with 10% FBS (Sigma). H22 cells were grown in RPMI-1640 (Gibco) supplemented with 10% FBS (GE Healthcare). Human PBMC or isolated CD3+T cells and Raji cells were cultured in RPMI-1640 (Gibco) with 10% FBS (Hyclone).

### Cell-based co-culture system using primary human CD3 + T cells and Raji cells

Functional activity of ipilimumab was evaluated by IL-2 production. Briefly, frozen human PBMCs were thawed and stimulated by 2.5 μg/mL Phytohemagglutinin-L (Thermo Fisher Scientific) for 6 days. CD3+cells were isolated by CD3 MicroBeads (Myltenyi Biotech) and co-cultured with engineered Raji cells, expressing an engineered cell surface protein designed to activate cognate TCRs in an antigen-independent manner and endogenously expressing CTLA-4 ligands CD80 and CD86, and ipilimumab mIgG2a or ipilimumab mIgG2a LALAPG for 24 h. Culture supernatants were collected, and IL-2 production was evaluated by human IL-2 DuoSet ELISA (R&D Systems) according to the manufacturer’s protocol.

### Generation of human CTLA-4 knock-in BALB/c mice via the CRISPR/Cas9 system

Adult human CTLA-4 knock-in BALB/c mice (generated through Taconic) were used for in vivo efficacy studies. The targeting strategy was based on NCBI transcripts NM009843.4 (mouse) and NM005214.4 (human). The targeting construct replaced mouse exons 2 and part of 3 with human exons 2 and part of 3. Potential off-targets were analyzed using the GRCm38/mm10 assembly. BALB/c ES cells were transfected with a plasmid expressing mammalian-codon-optimized Cas9 and sgRNA, plasmid containing a puromycin resistance cassette, and plasmid targeting mouse CTLA-4 and replaced with the human region. After drug selection, individual colonies were picked and genotyped by Southern blot analysis. Germline transmission of the replaced exons was verified by PCR analysis and Southern blotting. Protein expression was confirmed by cell surface and intracellular flow cytometry.

### Murine tumor models

Female C57BL/6 mice and human CTLA-4 knock-in C57BL/6 mice were inoculated subcutaneously with 1 × 10^6^ MC38 cells in the flank. Female BALB/c mice and human CTLA-4 knock-in BALB/c mice were inoculated subcutaneously with 4 × 10^5^ H22 cells in the flank. Tumor growth was monitored with vernier calipers, and the mean tumor volume was calculated using the formula [0.5 × (length × width^2^)]. When the mean tumor volume reached approximately 60 mm^3^, animals were randomized into treatment groups and dosing was initiated on Day 0 of the study. Tumor size and body weight were measured three times weekly. During the observation period, animals bearing oversized tumor exceeding 2000 mm^3^ were sacrificed. H11, H11-HLE, anti-mCTLA-4 Ig (9D9), Ipi-WT and Ipi-LALAPG were administered intravenously twice weekly for 3 weeks.

### Pharmacokinetic analysis

C57BL/6 mice bearing MC38 tumors were administered a single intravenous dose of H11 or H11-HLE at 30 mg/kg, and the tumor and plasma were harvested over a 120 h period. Human CTLA-4 knock-in C57BL/6 mice bearing MC38 tumors were administered a single intravenous dose of Ipi-WT or Ipi-LALAPG at 3 mg/kg, and the tumor and plasma were harvested over a 168 h period. Tumor tissue samples were homogenized with metal beads in a fourfold volume of the tissue protein extraction reagent containing 1% of a protease inhibitor cocktail by using a FastPrep-24 homogenizer. Drug exposures in plasma and tumor homogenate were determined using a ligand binding assay (LBA). LBA methods were developed and used in PK studies by using the MSD (Mesoscale discovery) platform. A biotinylated rabbit-anti-his tag antibody from Abcam was used for capture, and ruthenium-labeled human CTLA-4 was used as a detection reagent for H11 or H11-HLE by using an MSD streptavidin-coated plate. A recombinant-CTLA-4 protein was used for capture, and ruthenium-labeled goat anti-mouse IgG2a antibody from Abcam was used as a detection reagent for Ipi-WT or Ipi-LALAPG by using an MSD high bind plate. PK analysis of plasma and tumor concentration data was performed using Watson LIMS™ software, Version 7.5 (Thermo Scientific™ [Waltham, MA, the USA]). Kinetic parameters were estimated using a noncompartmental model. AUC was calculated using the linear trapezoidal rule.

### Pharmacodynamic analysis

Human CTLA-4 knock-in C57BL/6 mice bearing MC38 tumors were administered a single intravenous dose of Ipi-WT or Ipi-LALAPG at 3 mg/kg. The mice were euthanized 72 h after the single-dose administration, and the tumor was harvested for flow cytometry analysis.

### Flow cytometry

To prepare tissues for flow cytometry, tumor samples were digested by using a Mouse Tumor Dissociation Kit (Miltenyi). Red blood cells were lysed in Lysing Buffer (BD Bioscience). Cells were washed, pelleted and re-suspended in Stain buffer (BD Bioscience). For each sample, 5 × 10^6^ cells were treated with Live/Dead cell stain kit (Thermo Fisher). Cells were washed, pelleted and re-suspended in stain buffer containing Fc Block (BioLegend) and then stained with a defined panel containing various different labeling antibodies. All antibodies were purchased from BD Bioscience, BioLegend and Thermo Fisher as indicated. Data were measured on BD LSR Fortessa and analyzed by using FlowJo software. Flow cytometry antibodies used in this study were purchased from BD Bioscience [anti-mouse CD45 (30-F11), anti-mouse Ki67 (B56), anti-mouse CD8 (53-6.7), anti-mouse CD4 (GK1.5)], BioLegend [anti-mouse NKp46 (29A1.4), anti-mouse CD19 (1D3), anti-mouse Ly6G (1A8), anti-mouse CD62L (MEL-14), anti-mouse TCRb (H57-597), anti-mouse ICOS (C398.4A), anti-mouse CD25 (PC61.5), anti-mouse CD44 (IM7), anti-human CTLA-4 (BNI3), and Thermo Fisher anti-mouse Foxp3 (FJK-16S)].

### Statistics

Statistical analyses were performed using log-rank test and one-way ANOVA with Dunnett’s multiple comparisons test in GraphPad PRISM software.

## Results

### Generation of half-life extended anti-mouse CTLA-4 VHH

**Fig. 1:**
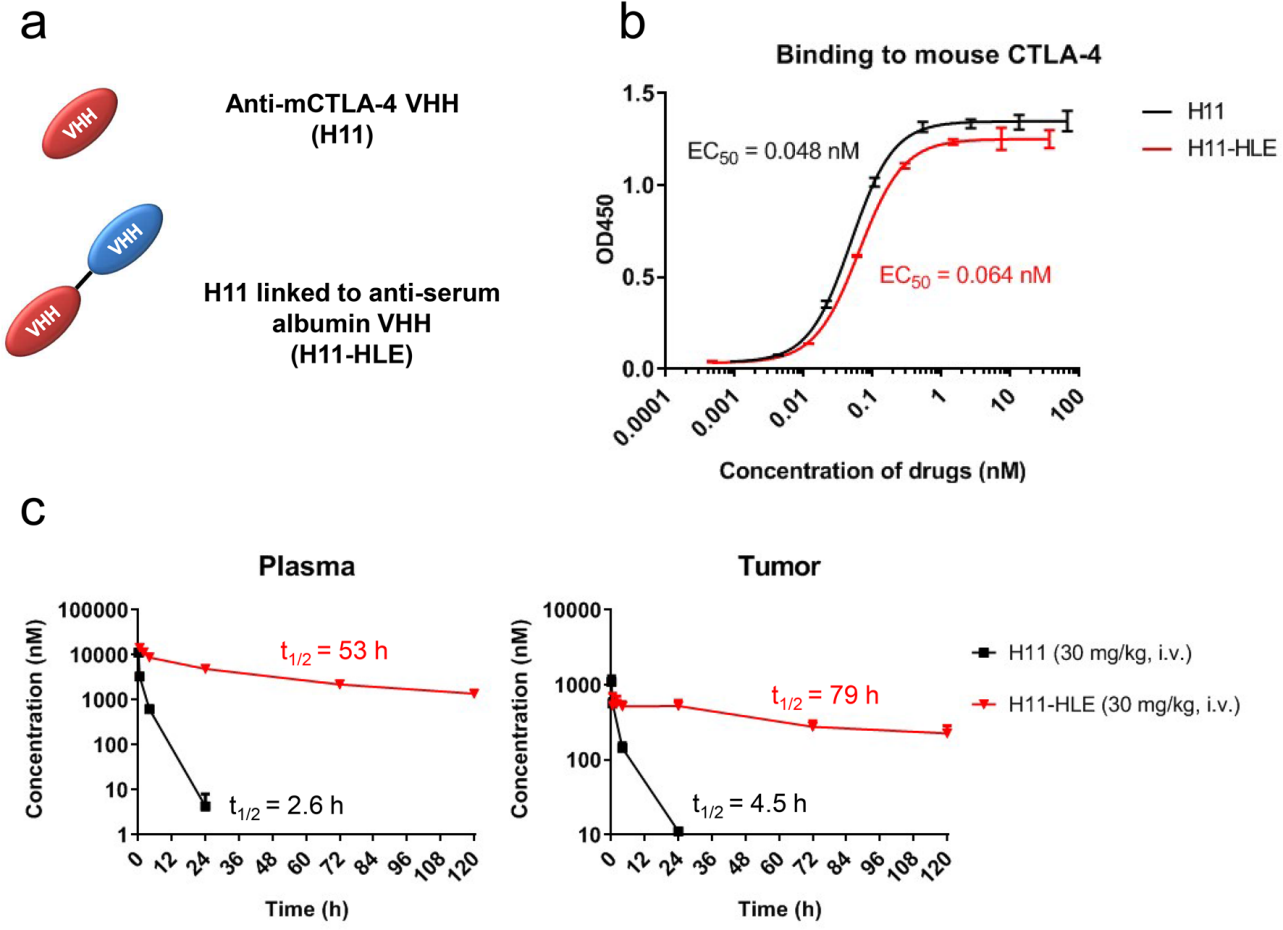
Half-life extended H11 shows similar binding to mouse CTLA-4 as H11 but longer half-life than H11. **a** Schematic representation of anti-mouse CTLA-4 VHH (H11) and H11 linked to anti-serum albumin VHH (H11-HLE). **b** Binding to mCTLA-4 of H11 and H11-HLE and their EC_50_ values. His-tagged H11 or H11-HLE was incubated with plate-bound mCTLA-4-Fc. Binding to mCTLA-4 was detected by using HRP-conjugated anti-His tag antibodies and tetramethylbenzidine (TMB). Data are represented as OD. Error bars show SD. **c** Plasma (left) and tumor (right) concentration–time profiles and half-life of H11 (30 mg/kg) or H11-HLE (30 mg/kg) in MC38 tumor-bearing C57BL/6 mice (*n* = 3) via intravenous administration. C57BL/6 mice were inoculated with 1 × 10^6^ MC38 cells. When the mean tumor volume reached approximately 300–500 mm^3^, animals were randomized into treatment groups (*n* = 3/group) and dosing was initiated. At indicated time points after single dosing, mice were euthanized and plasma and tumor tissues were harvested. H11 or H11-HLE was quantified using ELISA using HRP-conjugated anti-His tag antibodies

Previously, H11 was identified as a high-affinity alpaca heavy chain-only antibody fragment (VHH) against mouse CTLA-4 [14]. H11 binds mouse CTLA-4 and blocks the interaction between CTLA-4 and its ligand at lower concentrations than the widely used full-sized anti-mCTLA-4 antibody (9H10). Therefore, H11 is a useful tool molecule to evaluate the lack of the Fc function in immune checkpoint therapy, specifically blockade of CTLA-4 interaction with CD80/CD86. However, due to their small size, VHHs have rapid renal clearance and a much shorter half-life compared to conventional IgG antibodies [19]. To circumvent the decreased in vivo half-life, we generated an anti-mouse CTLA-4 VHH molecule with prolonged half-life, the H11 backbone protein was engineered with or without an anti-serum albumin VHH (referred subsequently in this report as H11-HLE) (Fig. 1a and Supplementary Fig. 1). As the addition of an HLE to H11 could potentially negatively impact CTLA-4 binding, we evaluated the binding affinity of H11 and H11-HLE in a cell-free plate-binding assay to recombinant mouse CTLA-4. Compared to H11, the half-life extended H11 maintained similar binding affinity to mouse CTLA-4 (Fig. 1b). To verify that H11-HLE exhibited improved in vivo half-life, we measured the pharmacokinetic (PK) profile of H11 and H11-HLE in MC38 tumor-bearing C57BL/6 mice via intravenous administration at 30 mg/kg. H11-HLE showed > tenfold longer half-life in plasma and tumor compared with non-half-life extended H11 in the tumor-bearing mice (Fig. 1c). Taken together, H11-HLE maintained similar binding affinity with improved in vivo half-life. Thus, H11-HLE is a tool molecule that allows the interrogation of the contribution of Fc in mediating immune checkpoint therapy.

### Half-life extended anti-mouse CTLA-4 VHH induced anti-tumor responses

**Fig. 2:**
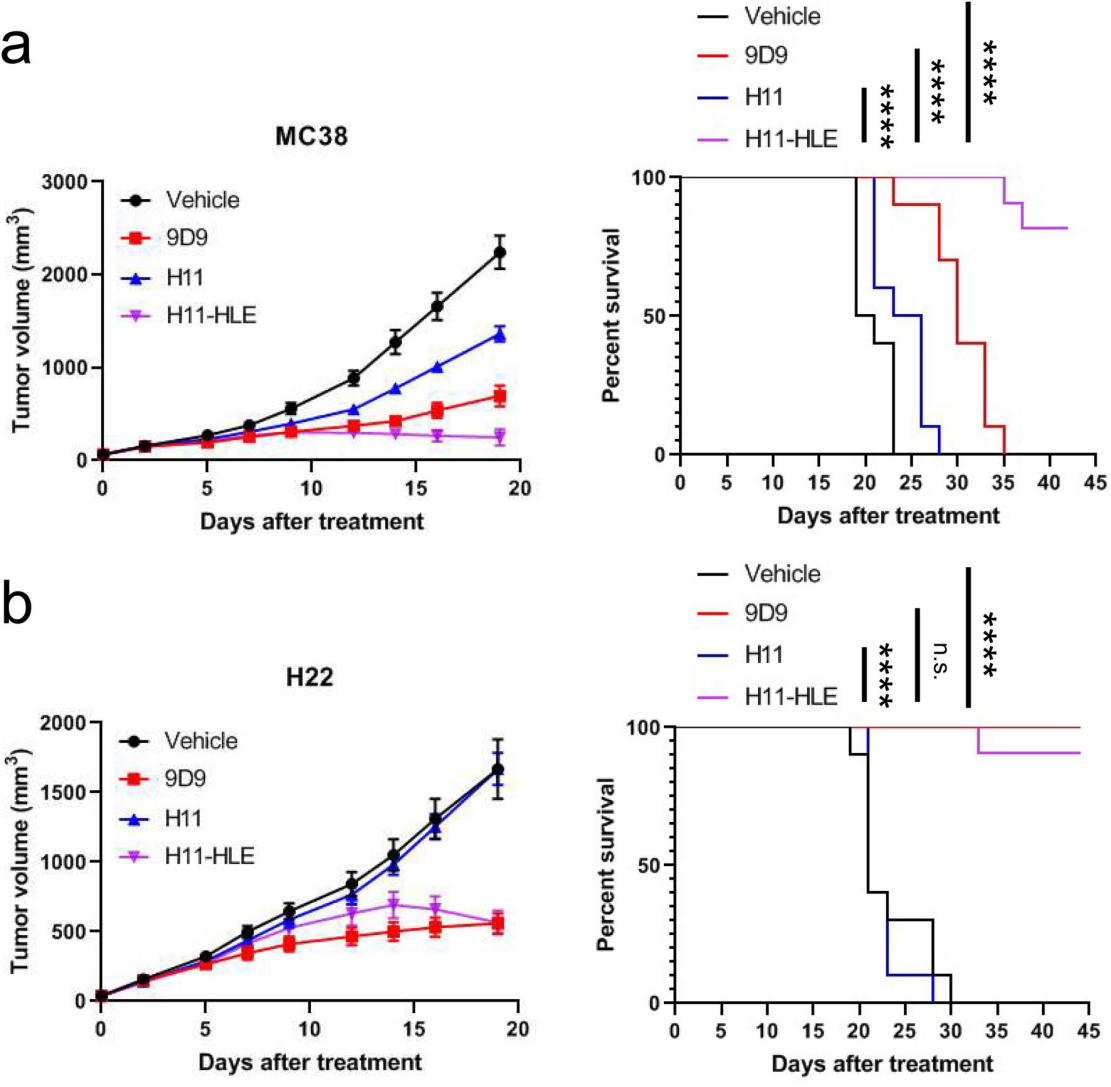
Half-life extended H11 induces anti-tumor efficacy in syngeneic tumor models. **a** C57BL/6 mice were inoculated with 1 × 10^6^ MC38 cells and were treated with PBS (vehicle), 10 mg/kg of anti-mCTLA-4 mIgG2b (9D9), 30 mg/kg of H11 and 30 mg/kg of H11-HLE twice weekly for 3 weeks. When the mean tumor volume reached approximately 60 mm^3^, animals were randomized into treatment groups (*n* = 10/group) and dosing was initiated on Day 0 of the study. **b** BALB/c mice were inoculated with 4 × 10^5^ H22 cells and were treated with PBS (vehicle), 10 mg/kg of anti-mCTLA-4 mIgG2b (9D9), 30 mg/kg of H11 and 30 mg/kg of H11-HLE twice weekly for 3 weeks. When the mean tumor volume reached approximately 60 mm^3^, animals were randomized into treatment groups (*n* = 10/group) and dosing was initiated on Day 0 of the study. (Left) Tumor size as measured by vernier calipers, and the data shown in all panels are the mean (*n* = 10/group) ± SEM. (Right) Survival curve comparing treatment groups. Mice were euthanized when tumors reached 2000 mm^3^. n.s., nonsignificant. ****P < 0.0001 by log-rank test

The MC38 cell line is a murine colon adenocarcinoma tumor derived from the C57BL/6 mouse, and the H22 cell line is a murine hepatoma tumor derived from the BALB/c mouse, and both cell lines are commonly used as syngeneic tumor models. To test whether anti-CTLA-4 VHH with or without half-life extender could show therapeutic effects in different syngeneic models, we evaluated anti-tumor effects of H11 and H11-HLE in MC38 and H22 tumor-bearing C57BL/6 mice and BALB/c mice, respectively. Twice weekly treatments with 30 mg/kg of H11 for 3 weeks showed minimum or no anti-tumor effect in the MC38 or H22 model (Fig. 2a, b). In contrast, treatment with 30 mg/kg of H11-HLE controlled tumor growth and achieved long-term survival in both models. The mice treated with H11-HLE showed longer survival than mice treated with the full-sized anti-mouse CTLA-4 mIgG2b antibody (clone 9D9) in the MC38 model (Fig. 2a, b). Therefore, we showed that non-Fc-containing CTLA-4 VHH retained the ability to induce robust anti-tumor activity in preclinical tumor models.

### Generation of ipilimumab mIgG2a with Fc effector functions and the mutated ipilimumab mIgG2a lacking Fc effector functions

The unexpected observation of potent anti-tumor activity mediated by H11-HLE, which is devoid of Fc-effector functions, prompted us to interrogate whether Fc effector functions are required for anti-tumor activity observed for ipilimumab, the only clinically approved anti-human CTLA-4 antibody. We generated anti-human CTLA-4 mIgG2a (Ipi-WT) with an intact Fc domain and an Fc mutant of ipilimumab with three amino acid substitutions in the Fc domain (L234A, L235A, P329G) (Ipi-LALAPG), thus eliminating any FcγR-dependent activities [20] (Supplementary Fig. 2).

**Fig. 3:**
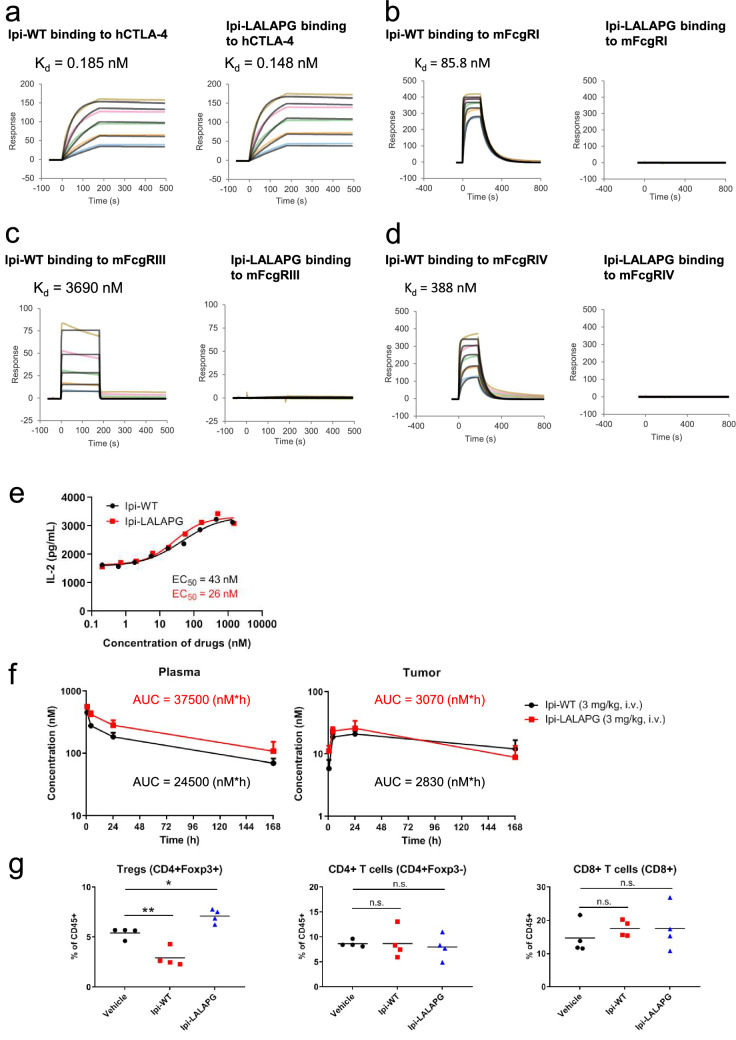
Ipilimumab mIgG2a LALAPG exhibits similar binding to human CTLA-4 and similar plasma and tumor PK as ipilimumab mIgG2a wildtype but does not bind to mouse FcγRs or induce in vivo Fc-dependent intratumoral Treg depletion. Surface plasmon resonance measurements of Ipi-WT and Ipi-LALAPG to **a** human CTLA-4, **b** mouse FcγRI, **c** FcγRIII and **d** FcγRIV. Human CTLA-4 was injected at concentrations ranging from 3.125 to 50 nM, and mouse FcγRs were injected at concentrations ranging from 187.5 to 3000 nM. **e** Inhibitory activity against interaction between CTLA-4 and B7 ligands by Ipi-WT and Ipi-LALAPG and their EC_50_ values. PHA-stimulated human primary CD3 + T cells expressing huCTLA-4 were co-cultured with engineered Raji cells expressing CTLA-4 ligands CD80 and CD86 in the presence of Ipi-WT or Ipi-LALAPG. Data are representative of 4 donors. Human-CTLA-4 knock-in C57BL/6 mice were inoculated with 1 × 10^6^ MC38 cells. When the mean tumor volume reached approximately 580 mm^3^, animals were randomized into treatment groups (*n* = 3/group) and dosing was initiated. **f** Plasma (left) and tumor (right) concentration–time profiles and the AUC of Ipi-WT (3 mg/kg) or Ipi-LALAPG (3 mg/kg) in MC38 tumor-bearing huCTLA-4 knock-in C57BL/6 mice (*n* = 3) via intravenous administration. Mice were treated with Ipi-WT or Ipi-LALAPG at day 0. At indicated time points after single dosing, mice were euthanized and plasma and tumor tissues were harvested. Ipi-WT or Ipi-LALAPG was quantified using ELISA. **g** Quantification of tumor-infiltrating Tregs, CD4 + T cells and CD8 + T cells. Mice (*n* = 4) were treated with PBS (vehicle), 3 mg/kg of Ipi-WT or 3 mg/kg of Ipi-LALAPG at day 0. The mice were euthanized 72 h after single dosing, and single-cells were isolated from resected tumors and analyzed by flow cytometry. n.s., nonsignificant. *P < 0.05 and **P < 0.01 by one-way ANOVA with Dunnett’s multiple comparisons test

We first evaluated the binding affinity of Ipi-WT and Ipi-LALAPG to human CTLA-4 and to several mouse FcγRs in a surface plasmon resonance (SPR) assay system. Compared with Ipi-WT, the SPR analysis showed that Ipi-LALAPG had similar binding affinity to huCTLA-4 (Fig. 3a). Ipi-WT also bound to mouse FcγRI, III, and IV, while Ipi-LALAPG did not exhibit binding to any of the FcγRs (Fig. 3b–d). Next, we evaluated the inhibitory activities of Ipi-WT and Ipi-LALAPG in a cell-based co-culture system using primary activated CD3 + T cells and engineered Raji cells expressing CTLA-4 ligands CD80 and CD86. Consistent with similar binding to huCTLA-4, Ipi-WT and Ipi-LALAPG enhanced IL-2 production with the similar EC_50_ values (Fig. 3e).

Next, to measure the PK profile of Ipi-WT and Ipi-LALAPG, we performed single dose PK studies at 3 mg/kg in MC38 tumor-bearing human CTLA-4 knock-in (huCTLA-4 KI) C57BL/6 mice. The huCTLA-4 KI mice have the extracellular domain of human CTLA-4 protein and intracellular domain of mouse CTLA-4 protein. There was no notable difference in plasma and tumor PK between Ipi-WT and Ipi-LALAPG (Fig. 3f).

In many preclinical murine tumor models, intratumoral Tregs were depleted via ADCC following treatment by different clones of anti-mCTLA-4 mAbs (clones 9D9, 9H10 and 4F10) [11–13, 15], similarly, ipilimumab hIgG1 also decreased tumor-infiltrating Tregs in the MC38 model [21]. To investigate the in vivo Fc-mediated intratumoral Treg depletion potential of Ipi-WT and Ipi-LALAPG, we performed single-dose pharmacodynamics (PD) studies in the MC38 tumor-bearing huCTLA-4 KI mice. Flow cytometry analysis showed that a single treatment of Ipi-WT selectively reduced intratumoral Tregs without affecting populations of conventional CD4 + T cells (non-Tregs) and CD8 + T cells, while Ipi-LALAPG did not decrease the Tregs (Fig. 3g). Taken together, consistent to previous reports, Ipi-WT (mIgG2a) molecule described here exhibited similar intratumoral Treg depletion, and this is in contrast to Ipi-LALAPG which lacked intratumoral Treg depletion activity.

### Ipilimumab lacking Fc effector functions can induce anti-tumor responses

**Fig. 4:**
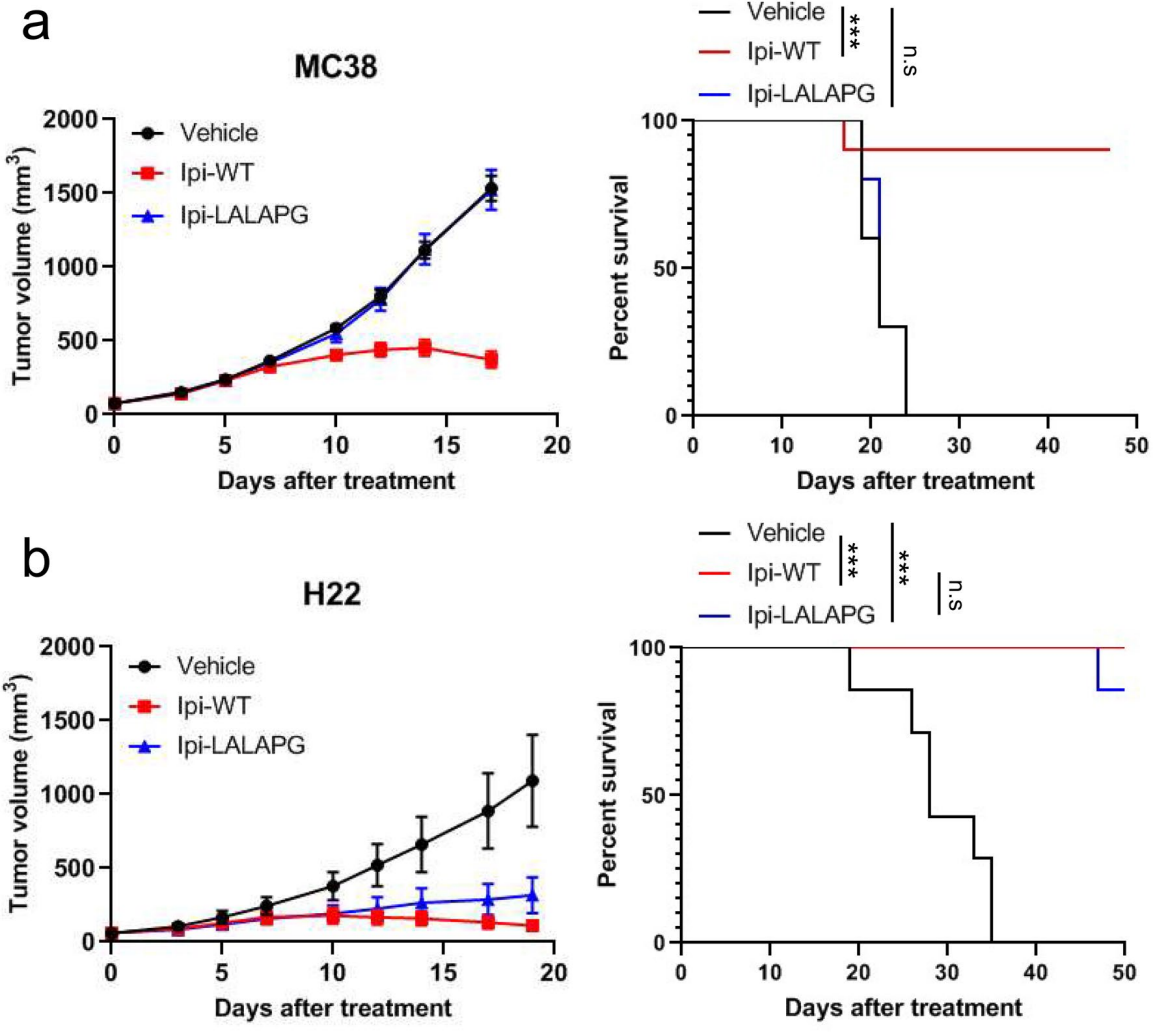
Ipilimumab lacking Fc effector functions induces anti-tumor responses. **a** HuCTLA-4 KI C57BL/6 mice were inoculated with 1 × 10^6^ MC38 cells and were treated with PBS (vehicle), 3 mg/kg of ipilimumab mIgG2a (Ipi-WT), or 3 mg/kg of ipilimumab mIgG2a LALAPG (Ipi-LALAPG) twice weekly for 3 weeks. When the mean tumor volume reached approximately 60 mm^3^, animals were randomized into treatment groups (*n* = 10/group) and dosing was initiated on Day 0 of the study. **b** HuCTLA-4 KI BALB/c mice were inoculated with 4 × 10^5^ H22 cells and were treated with PBS (vehicle), 3 mg/kg of ipilimumab mIgG2a LALAPG (Ipi-LALAPG) twice weekly for 3 weeks. When the mean tumor volume reached approximately 60 mm^3^, animals were randomized into treatment groups (*n* = 7/group) and dosing was initiated on Day 0 of the study. (Left) Tumor size as measured by vernier calipers, and the data shown in all panels are the mean (*n* = 7–10/group) ± SEM. (Right) Survival curve comparing treatment groups. Mice were euthanized when tumors reached 2000 mm^3^. n.s., nonsignificant. ***P < 0.001 by log-rank test

To further investigate the contribution of Fc effector functions to the therapeutic effect of ipilimumab in preclinical models, we treated MC38 tumor-bearing huCTLA-4 KI C57BL/6 mice and H22 tumor-bearing huCTLA-4 KI BALB/c mice (Supplementary Fig. 3) with Ipi-WT and Ipi-LALAPG. In the MC38 model, 3 mg/kg of Ipi-WT controlled the tumor growth and prolonged survival rate of the mice, while the same dose of Ipi-LALAPG had no observable anti-tumor effect (Fig. 4a). However, in the H22 model, Ipi-WT (3 mg/kg) also showed significant anti-tumor activity and Ipi-LALAPG (3 mg/kg) achieved similar tumor control and survival rate as Ipi-WT (Fig. 4b). Thus, we found that different syngeneic tumor models exhibited differential response to anti-CTLA-4 therapy in the presence or absence of Fc-effector functions.

## Discussion

Although previous studies demonstrated that anti-CTLA-4 therapy required Fc effector functions for the anti-tumor efficacy [11–14, 16], the data presented here, to our knowledge, revealed for the first time in preclinical syngeneic tumor models an Fc-independent mechanism for inducing anti-tumor immunity following treatment with anti-CTLA-4 modalities. This finding is consistent with previous work suggesting that CTLA-4 blockade of effector T cells and Tregs has an important role in the therapeutic effect of anti-CTLA-4 therapy [10].

H11 was previously identified as a VHH that binds to mouse CTLA-4 with high affinity and blocks in vitro interaction between CTLA-4 and B7-1 ligand [14]. H11 and the pegylated H11 with half-life extension showed minimal anti-tumor efficacy in combination with GVAX in the B16F10 model [14]. Lack of Fc effector functions of H11, leading to lack of intratumoral Treg depletion, was proposed as the reason for the minimal efficacy. However, the half-life and binding properties of the pegylated H11 were not examined. Therefore, possible reason for the minimal efficacy would be due to insufficient exposure of pegylated H11 and to negative impact by pegylation on the binding affinity of H11 to mouse CTLA-4. The other possible reason is that the B16F10 model was less sensitive to anti-CTLA-4 antibodies lacking Fc-effector functions than MC38 and H22 models used in the present study.

In this study, we generated H11 conjugated with an anti-serum albumin VHH and examined its potential in promoting anti-tumor immunity in other syngeneic models. We confirmed that conjugation of the half-life extender does not affect the binding affinity of H11 to mouse CTLA-4 and prolongs half-life in vivo. Despite the lack of an Fc, the half-life extended H11 showed robust anti-tumor efficacy in the MC38 and H22 models, and the activity is comparable to or even superior to anti-mCTLA-4 mIgG2b (clone 9D9) with Fc effector functions in the MC38 and H22 models. In contrast, H11 without a half-life extender showed minimal anti-tumor effect. Therefore, in contrast to the previously published data, our results show that longer half-life, and not Fc effector functions, is necessary and sufficient for the anti-tumor efficacy of H11 in these models. Also, based on its in vitro activity to block the interaction between CTLA-4 and B7 ligand [14], our data suggest that the anti-tumor efficacy of half-life extended H11 would be attributed to inhibition of the interaction between CTLA-4 and its ligands.

We also generated ipilimumab mIgG2a with Fc effector functions and the L234A, L235A, P329G (LALAPG) variant without effector functions. Previously, the LALAPG variant has been shown to eliminate complement binding and fixation as well as FcγR-dependent, antibody-dependent, cell-mediated cytotoxicity in both murine IgG2a and human IgG1 without affecting binding to a target protein or the PK profile [20]. As expected, we characterized that the amino acid changes in the Fc-region do not impact the binding affinity to human CTLA-4, blocking activity for the B7-CTLA-4 interaction and PK profile, but negate its binding to mouse FcγRs and Fc-dependent intratumoral Treg depletion. In the MC38 model, 3 mg/kg of Ipi-LALAPG did not induce any anti-tumor efficacy, while the same dose of Ipi-WT induced robust anti-tumor efficacy. This indicates that the anti-tumor efficacy by Ipi-WT would be driven by Fc effector functions in MC38 model.

In contrast, the H22 model was much more sensitive to Ipi-LALAPG than the MC38 model. These data show differential contributions of CTLA-4 blockade and Fc effector functions to preclinical efficacy. This may indicate that the non-Treg depleting activity of anti-CTLA-4 therapy could benefit cancer patients. Previously, the mechanism of tumor-specific Treg depletion was proposed by the presence of FcγR expressing cells and higher surface expression of CTLA-4 on Tregs in the tumor [12]. One hypothesis is that the different sensitivities might be driven by differences in expression of FcγRs and/or the presence of macrophages and NK cells or the presence of high surface CTLA-4 expressing Tregs in these tumor models. On the other hand, CTLA-4 blockade can enhance tumor-specific T cell priming mediated by antigen-presenting cells (APCs) expressing B7 ligands. Therefore, another hypothesis is that the different sensitivities might be affected by a difference in expression of B7-ligands and/or the presence of APCs expressing B7 ligands. Further studies are needed to clarify why the sensitivity to Ipi-LALAPG is different between these two models.

In the clinic, anti-CTLA-4 alone or in combination with other therapeutic agents is effective in a subset of cancer patients. However, systemic anti-CTLA-4 treatment frequently induces immune-related adverse events, (irAEs) and the underlying biological mechanisms are not fully understood yet [22]. Of particular interest, it is not clear if either (or both) Fc effector functions or Fc-independent functions, including CTLA-4 blockade of anti-CTLA-4 therapy, contribute to the irAEs in the clinic. In a clinically relevant model where anti-CTLA-4 therapy-induced irAEs are observed, our generated half-life extended anti-CTLA-4 VHH and Fc-mutated ipilimumab LALAPG will be useful tools to address this question. Also, if the irAEs are mainly dependent on Fc-effector functions, a non-Fc-containing CTLA-4 blocking strategy would be one of the next generation CTLA-4 therapies with a safer profile than ipilimumab.

### Supplementary Information

Below is the link to the electronic supplementary material.

Supplementary file1 (PDF 1148 kb)
