# Supplementary material for: Fc-independent functions of anti-CTLA-4 antibodies contribute to anti-tumor efficacy
Source: Cancer Immunol Immunother. 2022 Mar 3;71(10):2421–31. doi: 10.1007/s00262-022-03170-z (PMC9463231; doi:10.1007/s00262-022-03170-z)
Supplement: Supplementary file 1 — Supplementary file1 (PDF 1148 kb) [file 262_2022_3170_MOESM1_ESM.pdf]

a

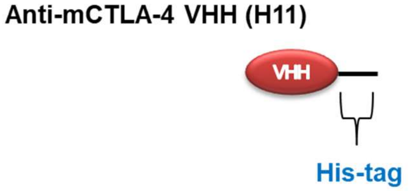

His tag: highlighted in blue
Signal Sequence: highlighted in red

MKYLLPTAAAGLLLLAAQPAMAQVQLVESGGGLAQP
GGSLRLSCAASGSTISSVAVGWYRQTPGNQREWVA
TSSTSSTTATYADSVKGRFTISRDNAKNTIYLQMNSL
KPEDTAVYYCKTGLTNWGRGTQVTVSSAAAHHHHH
HHHHH

Molecular weight: 13.6 kDa

b

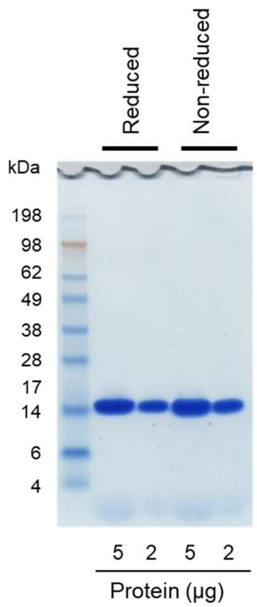

c

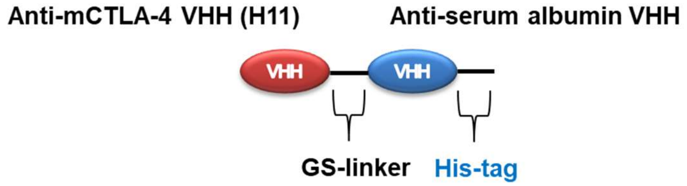

His tag: highlighted in blue
Signal Sequence: highlighted in red
Linker: highlighted in purple

METDTLLLWVLLLWVPGSTGQVQLVESGGGLAQPGGSL
RLSCAASGSTISSVAVGWYRQTPGNQREWVATSSTSSTT
ATYADSVKGRFTISRDNAKNTIYLQMNSLKPEDTAVYYCK
TGLTNWGRGTQVTVSSGGSGGSGGSGGSGGSGGSGG
SGAVQLVESGGGLVQPGNSLRLSCAASGFTFRSFGMSW
VRQAPGKEPEWVSSISGSGSDTLYADSVKGRFTISRDNA
KTTLYLQMNSLKPEDTAVYYCTIGGSLSRSSQGTQVTVS
SAAAHHHHHHHHHH

Molecular weight: 27.2 kDa

d

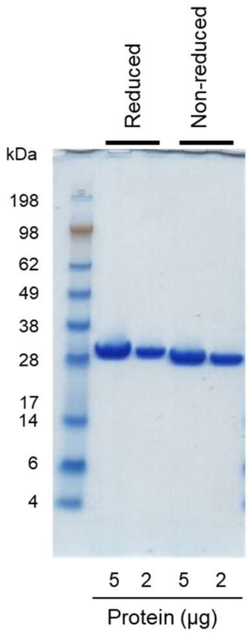

Supplementary Fig. 1 Generation of anti-mouse CTLA-4 VHH (H11) and half-life extended H11 linked to anti-serum albumin VHH (H11-HLE). Amino acid sequence and SDS-PAGE of H11 (**a** and **b**) and H11-HLE (**c** and **d**)

a

Signal Sequence: highlighted in red

Ipilimumab mIgG2a heavy chain
MGWSCIILFLVATATGVHSQVQLVESGGGVVQPGRSLRLSCAAS
GFTFSSYTMHWVRQAPGKGLEWVTFISYDGNNKYYADSVKGRF
TISRDNSKNTLYLQMNSLRAEDTAIYYCARTGWLGPFDYWGQGT
LVTVSSAKTTAPSVYPLAPVCGDTTGSSVTLGCLVKGYFPEPVTL
TWNSGSLSSGVHTFPAVLQSDLYTLSSSVTVTSSTWPSQSITCNV
AHPASSTKVDKKIEPRGPTIKPCPPCKCPAPNLLGGPSVFIFPPKI
KDVLMISLSPIVTCVVVDVSEDDPDVQISWFVNNVEVHTAQTQTH
REDYNSTLRVVSALPIQHQDWMSGKEFKCKVNNKDLPAPIERTI
SKPKGSVRAPQVYVLPPPEEEMTKKQVTLTCMVTDFMPEDIYVE
WTNNGKTELNYKNTEPVLDSDGSYFMYSKLRVEKKNWVERNSY
SCSVVHEGLHNHHTTKSFSRTPGK

Ipilimumab mIgG2a light chain
MGWSCIILFLVATATGVHSEIVLTQSPGTLSLSPGERATLSCRASQ
SVGSSYLAWYQQKPGQAPRLLIYGAFSRATGIPDRFSGSGSGTD
FTLTISRLEPEDFAVYYCQQYGSSPWTFGQGTKVEIKRADAAPTV
SIFPPSSEQLTSGGASVVCFLNNFYPKDINVKWKIDGSERQNGVL
NSWTDQDSKDSTYSMSSTLTLTKDEYERHNSYTCEATHKTSTSPI
VKSFNRNEC

Molecular weight: 146 kDa

b

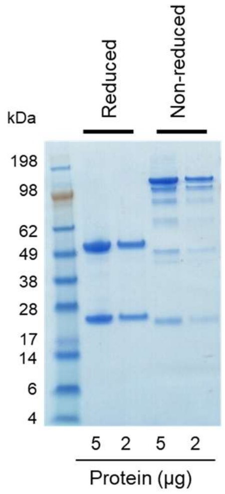

c

Signal Sequence: highlighted in red
Mutation in Fc region: highligyed in blue

Ipilimumab mIgG2a L234A_L235A_P329G heavy chain
MGWSCIILFLVATATGVHSQVQLVESGGGVVQPGRSLRLSCAAS
GFTFSSYTMHWVRQAPGKGLEWVTFISYDGNNKYYADSVKGRF
TISRDNSKNTLYLQMNSLRAEDTAIYYCARTGWLGPFDYWGQGT
LVTVSSAKTTAPSVYPLAPVCGDTTGSSVTLGCLVKGYFPEPVTL
TWNSGSLSSGVHTFPAVLQSDLYTLSSSVTVTSSTWPSQSITCNV
AHPASSTKVDKKIEPRGPTIKPCPPCKCPAPNAAGGPSVFIFPPKI
KDVLMISLSPIVTCVVVDVSEDDPDVQISWFVNNVEVHTAQTQTH
REDYNSTLRVVSALPIQHQDWMSGKEFKCKVNNKDLGAPIERTI
SKPKGSVRAPQVYVLPPPEEEMTKKQVTLTCMVTDFMPEDIYVE
WTNNGKTELNYKNTEPVLDSDGSYFMYSKLRVEKKNWVERNSY
SCSVVHEGLHNHHTTKSFSRTPGK

Ipilimumab mIgG2a L234A_L235A_P329G light chain
MGWSCIILFLVATATGVHSEIVLTQSPGTLSLSPGERATLSCRASQ
SVGSSYLAWYQQKPGQAPRLLIYGAFSRATGIPDRFSGSGSGTD
FTLTISRLEPEDFAVYYCQQYGSSPWTFGQGTKVEIKRADAAPTV
SIFPPSSEQLTSGGASVVCFLNNFYPKDINVKWKIDGSERQNGVL
NSWTDQDSKDSTYSMSSTLTLTKDEYERHNSYTCEATHKTSTSPI
VKSFNRNEC

Molecular weight: 146 kDa

d

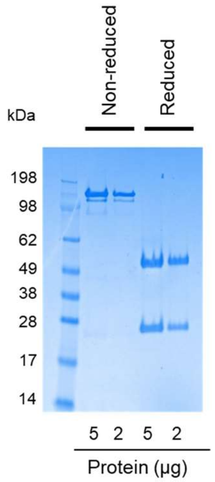

Supplementary Fig. 2 Generation of Ipilimumab mIgG2a (Ipi-WT) and Ipilimumab mIgG2a LALAPG (Ipi-LALAPG). Amino acid sequence and SDS-PAGE of Ipi-WT (**a** and **b**) and Ipi-LALAPG (**c** and **d**)

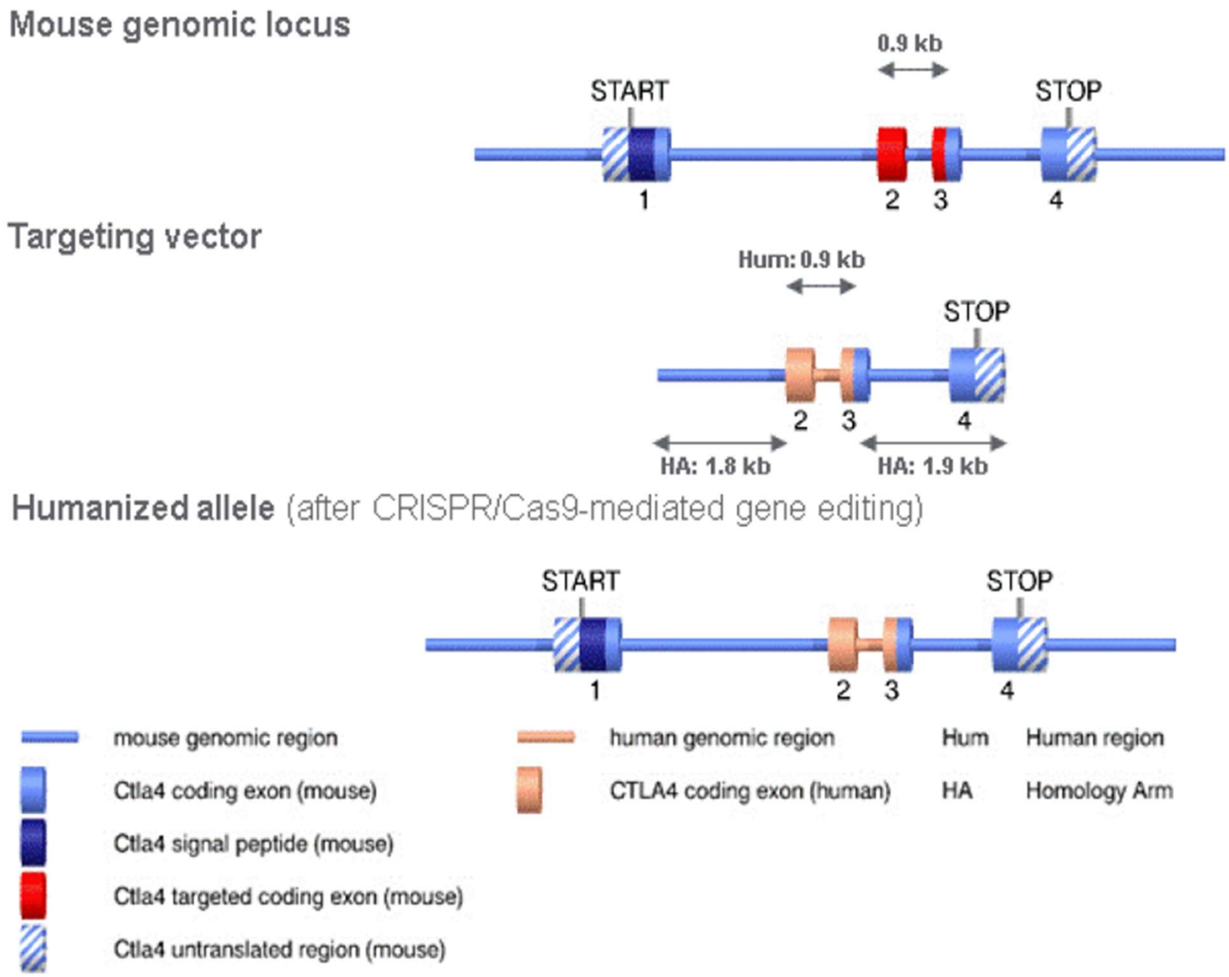

Supplementary Fig. 3 Humanization of the Ctla4 gene via CRISPR/Cas9-mediated gene editing
The targeting construct replaced the exons coding for the extracellular (exon 2) and part of transmembrane (exon 3) domains of murine CTLA-4 with those of human CTLA-4.
